# Has the role of veno-arterial extracorporeal membrane oxygenation in patients with cardiogenic shock following acute myocardial infarction been fully determined? A case report

**DOI:** 10.1093/ehjcr/ytae125

**Published:** 2024-03-12

**Authors:** Kha Minh Nguyen, Hai Phuong Nguyen Tran, Vi Tuong Dang, Sy Van Hoang

**Affiliations:** Department of Internal Medicine, Faculty of Medicine, University of Medicine and Pharmacy at Ho Chi Minh City, No. 217, Hong Bang Street, Ward 11, District 5, Ho Chi Minh City 700000, Vietnam; Department of Cardiology, Cho Ray Hospital, No. 201B, Nguyen Chi Thanh Street, Ward 12, District 5, Ho Chi Minh City 700000, Vietnam; Department of Interventional Cardiology, Cho Ray Hospital, Ho Chi Minh City, Vietnam; Department of Internal Medicine, Faculty of Medicine, University of Medicine and Pharmacy at Ho Chi Minh City, No. 217, Hong Bang Street, Ward 11, District 5, Ho Chi Minh City 700000, Vietnam; Department of Internal Medicine, Faculty of Medicine, University of Medicine and Pharmacy at Ho Chi Minh City, No. 217, Hong Bang Street, Ward 11, District 5, Ho Chi Minh City 700000, Vietnam; Department of Cardiology, Cho Ray Hospital, No. 201B, Nguyen Chi Thanh Street, Ward 12, District 5, Ho Chi Minh City 700000, Vietnam

**Keywords:** Acute myocardial infarction, Cardiogenic shock, VA ECMO, Percutaneous coronary intervention, Case report

## Abstract

**Background:**

The persistent challenge of high mortality rates in acute myocardial infarction–induced cardiogenic shock endures notwithstanding advancements in the diagnosis and treatment of this disease over the past two decades. While recent studies present conflicting evidence on the efficacy of veno-arterial extracorporeal membrane oxygenation (VA ECMO), observational research supports the benefits of early VA ECMO initiation. However, the current lack of robust support from randomized clinical trials for VA ECMO use in this context highlights the ongoing uncertainty surrounding its effectiveness.

**Case summary:**

A 52-year-old male with sudden, intense chest pain was diagnosed with cardiogenic shock due to non–ST-elevation acute myocardial infarction at a local hospital. Initial treatment included aspirin, clopidogrel, and noradrenaline. Upon transfer to our hospital, the patient’s condition deteriorated, leading to acute respiratory distress and severe hypotension. Prior to emergent percutaneous coronary intervention, peripheral VA ECMO was initiated. Coronary angiography revealed left main coronary artery occlusion, and a successful intervention was performed. Post-intervention, the patient’s haemodynamic parameters significantly improved, and after 7 days, ECMO was successfully discontinued. The patient was discharged in stable condition after 25 days, with favourable outcomes persisting at the 30-day mark. Continuous monitoring is planned during outpatient follow-up.

**Discussion:**

The clinical case illustrates a successful treatment outcome achieved through teamwork by the heart team, supporting the efficacy of the VA ECMO pre–percutaneous coronary intervention approach. The careful selection of appropriate candidates and strategic initiation of VA ECMO may play a role in enhancing outcomes for individuals experiencing acute myocardial infarction complicated by challenging cardiogenic shock.

**Learning points**

- Uncertain veno-arterial extracorporeal membrane oxygenation (VA ECMO) efficacy: recent studies present conflicting evidence on the effectiveness of VA ECMO in acute myocardial infarction–induced cardiogenic shock.
- Positive outcome with VA ECMO pre–percutaneous coronary intervention: timely initiation of VA ECMO, coupled with collaborative efforts by the heart team, emphasizes the importance of strategic patient selection and early intervention with VA ECMO.

## Introduction

Cardiogenic shock (CS) remains the most common cause of mortality in patients admitted for acute myocardial infarction (AMI), and the mortality rate has remained nearly unchanged over the past two decades.^1^ Although some recent studies have reported failures in demonstrating the role of veno-arterial extracorporeal membrane oxygenation (VA ECMO) in improving in-hospital outcomes,^2,3^ recent observational studies still indicate that that early initiation of VA ECMO provides benefits to patients.^4,5^ In the context of the role of mechanical circulatory support devices, especially VA ECMO, in AMI, short-term mechanical circulatory support for refractory CS patients is recommended as a bridge to myocardial recovery after revascularization.^1^ Acute myocardial infarction complicated by CS is a severe cardiac event associated with a high mortality rate of ∼50%, regardless of the potential benefits of early percutaneous coronary intervention (PCI).^6^ Following the publication of the ECLS-SHOCK study in 2023, this treatment approach continues to face significant challenges.^3^ The investigation into the effectiveness of intervention for a specific subset of AMI-complicated CS intrigues physicians in cardiac intensive care. This report details the successful management of a case at a tertiary referral hospital in Vietnam. Through a comparative analysis with existing literature, our objective is to identify factors that can enhance the efficacy of VA ECMO treatment in patients experiencing CS following AMI.

## Summary figure

**Table:** 

Date	Event
25 October 2023	Hospital admission at the local hospital due to chest pain.
	Diagnosis: Non–ST-elevation myocardial infarction (NSTEMI) with cardiogenic shock, very high-risk on the 4th hour.
	Transfer to our hospital.
26 October 2023 12:41 a.m.	Clinical condition worsened with decreased blood pressure, high-dose vasopressors, and cognitive impairment.
	Electrocardiogram at emergency department: acute anterior ST-elevation myocardial infarction (STEMI).
	Diagnosis: refractory cardiogenic shock—STEMI, extensive anterior infarct, and Killip IV.
26 October 2023 3:22 a.m.	Transfer to the cath lab.
	Consultation with the heart team.
	Initiation of veno-arterial extracorporeal membrane oxygenation (VA ECMO) before.
	Percutaneous coronary intervention with a drug-eluting stent in the left main coronary artery and left anterior descending I.
26 October–1 November 2023	Veno-arterial extracorporeal membrane oxygenation support in the coronary care unit.
1 November 2023	Successful weaning off VA ECMO and discontinuation of ECMO support.
	Transfer to the operating department for ECMO cannula removal.
1–14 November 2023	Continuation of optimal medical therapy.
14 November 2023	Discharged from our hospital.
	Follow-up scheduled at the outpatient cardiology clinic.

## Case presentation

**Figure 1:**
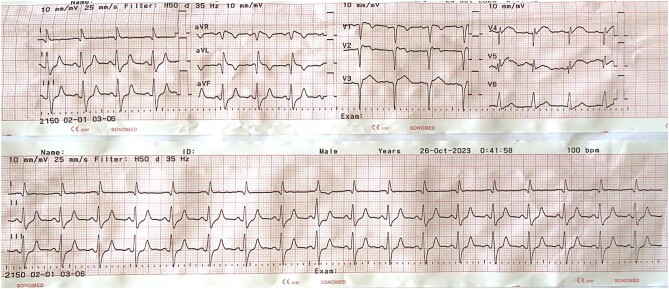
The 12-lead electrocardiogram at hospital admission.

**Figure 2:**
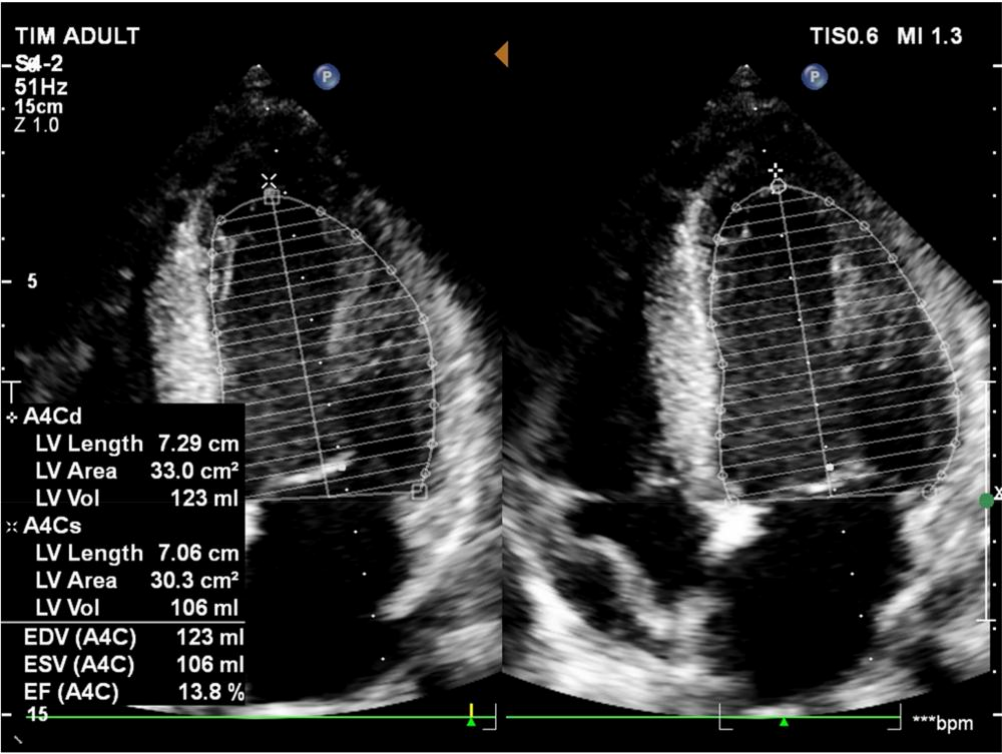
Echocardiography revealed a global reduction in left ventricular function, with an ejection fraction (EF) of 14%.

A 52-year-old male patient was admitted within 4 h of the onset of chest pain. Initial admission occurred at a local hospital, where the diagnosis of CS due to AMI was made. He denied any serious medical condition and is currently still smoking 20 pack-years. The patient received treatment, including aspirin 324 mg, clopidogrel 300 mg, enoxaparin 30 mg IV (initial dose) followed by 60 mg SC (total daily dose), and noradrenaline at 0.49 μg/kg/min, before being transferred to our centre. Upon admission, his presentation included pallor, cold extremities, a heart rate of 110 b.p.m., a blood pressure reading of 90/60 mmHg with the administration of noradrenaline, an elevated respiratory rate of 30 breaths per minute, and an oxygen saturation level of 94% while receiving oxygen through a 10 L per minute mask. The patient’s condition further deteriorated, presenting severe hypotension requiring an escalation of vasopressor therapy. The emergency electrocardiogram (ECG) revealed ST-segment elevation in leads V1–V4 and aVR (*Figure 1*). The echocardiography revealed global hypokinesis of the left ventricular ejection fraction (LVEF) at 14% (*Figure 2*). Laboratory findings indicated a significantly elevated hs troponin I level of 1286.1 ng/L (reference range: <34). Arterial blood gas analysis revealed a mixed acid–base disorder with a pH of 7.093, PaCO_2_ of 43.8 mmHg, HCO_3_^−^ of 13.4 mmol/L, and PaO_2_ of 95 mmHg. Other test results were presented in *Table 1*.

**Table 1:** Laboratory tests conducted on the patient at initial hospital admission

Parameters	Values	Reference range
Haemoglobin (g/L)	123	120–170
WBC (g/L)	12.2	4–11
PLT (g/L)	128	200–400
Glycaemia (mmol/L)	9.65	3.88–6.10
BUN (mmol/L)	8.57	2.50–7.14
Serum creatinine (mg/dL)	1.17	0.7–1.5
eGFR (CKD-EPI) (mL/min/1.73 m^2^)	71.2	≥90
SGOT (U/L)	183	9–48
SGPT (U/L)	57	5–49
CK-MB (U/L)	201.5	<25
hs Troponin I (ng/L)	12 861.1	<34
Na^+^ (mmol/L)	138	135–145
K^+^ (mmol/L)	4.5	3.5–4.5
Cl^−^ (mmol/L)	107	98–106
Lactate (Mmol/L)	5.05	0.5–2.2

WBC, white blood cell; PLT, platelet count; BUN, blood urea nitrogen; eGFR, estimated glomerular filtration rate; CKD-EPI, Chronic Kidney Disease Epidemiology Collaboration; SGOT, serum glutamic-oxaloacetic transaminase; SGPT, serum glutamic-pyruvic transaminase; CK-MB, creatine kinase-MB.

**Figure 3:**
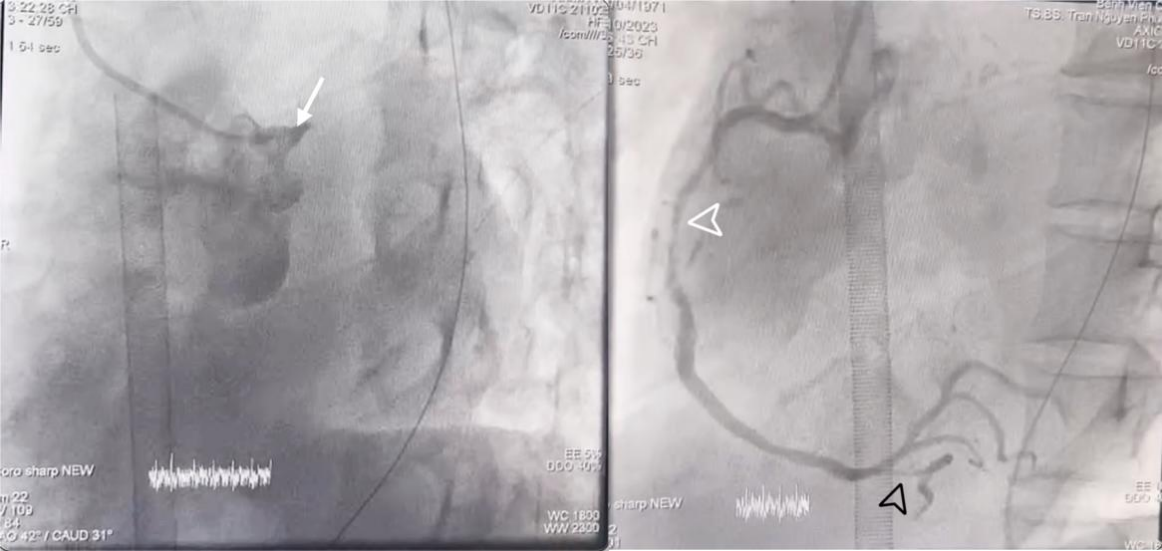
Results of the coronary angiogram. White arrowhead, total occlusion of the left main coronary artery; white arrowhead tip, 70–80% stenosis of the mid-right coronary artery (RCA II); and black arrowhead tip, 80% stenosis of the posterior descending artery (PDA).

The confirmed diagnosis by the 6th hour was ST-elevation myocardial infarction progressing to Killip IV, accompanied by Society for Cardiovascular Angiography and Interventions (SCAI) D–CS, indicative of acute decompensated heart failure. Following the consensus of the heart team, the patient underwent peripheral VA ECMO through femoral veno-arterial cannulation in the catheterization lab within 30 min before emergent PCI. The vasoactive-inotropic score (VIS) of the patient at the time of initiating VA ECMO was calculated to be 164 points. Coronary angiography revealed total occlusion of the left main coronary artery (LMCA), 70–80% stenosis of the mid-right coronary artery, and 80% stenosis of the posterior descending artery (*Figure 3*; Supplementary material online, *Video S1*). The LMCA–LAD I was intervened with a 3.0 × 4 mm stent, achieving TIMI III flow (see Supplementary material online, *Video S2*).

**Figure 4:**
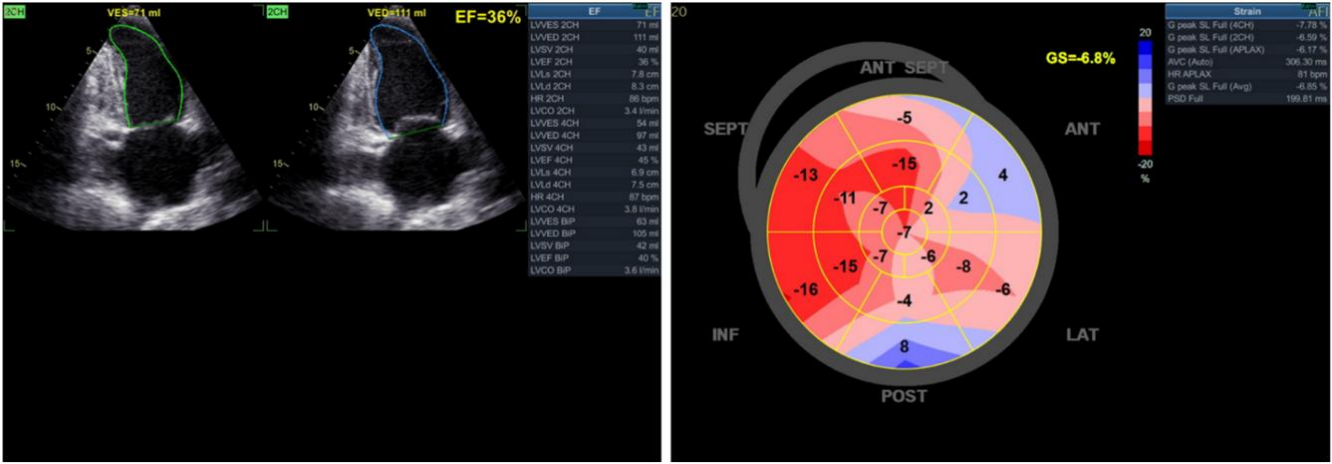
Echocardiography revealed an improvement in left ventricular contractile function (increasing from 14% to 36%). However, the global longitudinal strain (GLS) index on speckle tracking echocardiography remained low at −6.8%, suggesting injury in the anterior wall.

After a successful PCI, the patient was transferred to the coronary care unit for ongoing recovery. Subsequently, the patient’s haemodynamic status significantly improved, maintaining an average blood pressure of 78 mmHg and a noradrenaline infusion rate of 0.2 μg/kg/min. Vasopressor doses were gradually tapered and ceased after 6 h with VA ECMO. Continuous monitoring and maintenance on VA ECMO, coupled with advanced resuscitative measures, ensued. After 7 days, a notable improvement in cardiac function was observed, reflected in an LVEF of 36% (Simpson) and a global longitudinal strain (GLS) of 6.8% on speckle tracking echocardiography (*Figure 4*; Supplementary material online, *Video S3*). After a 25-day hospitalization, the patient was discharged in a haemodynamically stable condition with the following discharge medications: ticagrelor 90 mg BID, aspirin 81 mg OD, rosuvastatin 20 mg OD, ivabradine 5 mg BID, verospiron 25 mg OD, and sacubitril/valsartan 50 mg BID. After over 30 days post-discharge, two successful outpatient follow-up visits indicate the patient’s prognosis remains positive.

## Discussion

In the ECLS-SHOCK study, nearly half of the patients died, regardless of VA ECMO support. The 30-day all-cause mortality was 47.8% in the VA ECMO group and 49% in the control group.^3^ Notably, only 5.8% of VA ECMO recipients underwent left ventricular unloading, compared with 31.6% in the standard care group, where 14% received other mechanical circulatory support. In contrast, VA ECMO elevates afterload, presenting physiological drawbacks in the progression of CS due to AMI.^7,8^ Furthermore, 78% of the study patients underwent ECMO after CPR, and the timing of VA ECMO initiation was not clearly described. These characteristics pose challenges in demonstrating the actual benefits of VA ECMO in patients with complications of CS. A significant observation is the high incidence of left ventricular support device implantation, particularly Impella CP (85.7%) in the control group. The review by Russo *et al.* examined the role of implantation of percutaneous ventricular assistance devices in clinically indicated high-risk patients undergoing PCI and patients with CS or refractory cardiac arrest. In patients with severe CS due to AMI, the choice between Impella and VA ECMO may be considered. Additionally, for the purpose of left ventricular unloading, a combination of Impella + ECMO or intra-aortic balloon pump (IABP) + ECMO has shown promising evidence.^9^ Reports suggest that Impella’s role is comparable with VA ECMO in AMI-complicated CS, but accessibility remains an issue in developing countries like Vietnam.^10^ Moreover, the ECLS-SHOCK study did not report the VIS—a highly relevant index for determining the need for VA ECMO and predicting patient outcomes.^11^ The VIS is a numeric index used to measure cardiovascular support in shock patients, with higher scores indicating a more severe condition and poorer prognoses. In our case, the patient, aged 52, was younger than the average age in this study (63) and underwent VA ECMO intervention before PCI.

The optimal timing for initiating VA ECMO in comparison with PCI for patients experiencing CS due to AMI is a subject of thorough investigation. Current studies indicate that initiating VA ECMO before revascularization leads to significantly improved short- and long-term outcomes compared with cases where VA ECMO insertion occurs after revascularization.^12^ In a study of 253 CS patients supported by VA ECMO, initiating VA ECMO before revascularization significantly reduced major adverse events compared with ECMO after revascularization.^5^ Concerns persist about potential delays in revascularization for VA ECMO patients, a crucial treatment for reducing mortality. Huang *et al.*^4^ reported that VA ECMO before PCI in STEMI patients was linked to increased door-to-balloon time, although not statistically significant. Nevertheless, it significantly improved 6-month and 2-year survival rates, resulting in a significantly lower in-hospital mortality rate in that group.

The 2023 European Society of Cardiology guideline recommends considering short-term mechanical circulatory support for severe/refractory CS in AMI patients (class IIb).^13^ This report presents the first documented use of VA ECMO before PCI in a Vietnamese AMI case, resulting in positive outcomes. Cho Ray Hospital, known for ECMO expertise, marks its initial deployment of VA ECMO pre-PCI in such cases.^14^ Further evidence from larger, multicentre studies involving PCI patients is crucial to comprehensively understand VA ECMO’s role in AMI with CS.

## Conclusion

The clinical case we report is a successful treatment outcome achieved through the coordinated efforts of the heart team, providing additional evidence for the effectiveness of the VA ECMO before the PCI strategy. The judicious selection of suitable patients and optimal timing for initiating VA ECMO may contribute to improving outcomes for patients with AMI complicated by refractory CS.

## Supplementary Material

ytae125_Supplementary_Data

## Data Availability

The data underlying this article are available in the article and in its online Supplementary material.

## Lead author biography

Kha Minh Nguyen is currently a lecturer in the Department of General Internal Medicine, Faculty of Medicine, University of Medicine and Pharmacy at Ho Chi Minh City. He graduated from the internal medicine residency programme under the guidance of Associate Professor Sy Van Hoang at the University of Medicine and Pharmacy at Ho Chi Minh City. In addition, he has been practising as a cardiologist at Cho Ray Hospital (a national-level healthcare centre in Vietnam) since 2019, specializing in the diagnosis and treatment of acute coronary syndrome and heart failure.

## Notes

**Consent:** The authors confirm that written consent for submission and publication including images and texts has been obtained from the patient in line the Committee on Publication Ethics (COPE) guidance and under the review board of the hospital administration.

**Funding:** None declared.
